# Understanding the Distance Effect of the Single‐Atom Active Sites in Fenton‐Like Reactions for Efficient Water Remediation

**DOI:** 10.1002/advs.202307151

**Published:** 2024-01-15

**Authors:** Shuaiqi Zhang, Zhicong Lu, Chun Hu, Fan Li

**Affiliations:** ^1^ Key Laboratory for Water Quality and Conservation of the Pearl River Delta Ministry of Education Institute of Environmental Research at Greater Bay Guangzhou University Guangzhou 510006 China

**Keywords:** contaminant removal, distance effect, electron transfer regime, Fenton‐like reactions, single‐atom catalysts

## Abstract

Emerging single‐atom catalysts (SACs) are promising in water remediation through Fenton‐like reactions. Despite the notable enhancement of catalytic activity through increasing the density of single‐atom active sites, the performance improvement is not solely attributed to the increase in the number of active sites. The variation of catalytic behaviors stemming from the increased atomic density is particularly elusive and deserves an in‐depth study. Herein, single‐atom Fe catalysts (Fe_SA_‐CN) with different distances (*d*_site_) between the adjacent single‐atom Fe sites are constructed by controlling Fe loading. With the decrease in *d*_site_ value, remarkably enhanced catalytic activity of Fe_SA_‐CN is realized via the electron transfer regime with peroxymonosulfate (PMS) activation. The decrease in *d*_site_ value promotes electronic communication and further alters the electronic structure in favor of PMS activation. Moreover, the two adjacent single‐atom Fe sites collectively adsorb PMS and achieve single‐site desorption of the PMS decomposition products, maintaining continuous PMS activation and contaminant removal. Moreover, the Fe_SA_‐CN/PMS system exhibits excellent anti‐interference performance for various aquatic systems and good durability in continuous‐flow experiments, indicating its great potential for water treatment applications. This study provides an in‐depth understanding of the distance effect of single‐atom active sites on water remediation by designing densely populated SACs.

## Graphical abstract

The proximity of the adjacent Fe atoms can alter the electronic structure of individual Fe atoms and collectively adsorb PMS molecules with a two‐site pathway. The PMS decomposition products achieve single‐site desorption from the individual Fe atoms. Therefore, the distance effect between single‐atom Fe sites facilitates efficient contaminant remediation through an electron transfer regime.

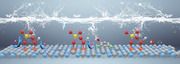

## 1 Introduction

In the last few decades, the imperative crisis of clean water scarcity resulting from emerging contaminant pollution has stimulated the innovation of water treatment technologies.^[^ ^1^ ^]^ Peroxymonosulfate (PMS)‐based Fenton‐like reaction is regarded as a promising advanced oxidation process (AOPs) to deal with the ever‐growing environmental pollution, relying on the generation of hydroxyl radicals (^●^OH) and sulfate radicals (SO_4_^●−^) with high redox potentials upon the cleavage of O─O bond in PMS (HSO_5_^−^).^[^ ^2^ ^]^ Over the past few years, transition metal (TM) catalysts (either in the form of ions or metal oxides) have served as highly efficient activators of the Fenton‐like reaction.^[^ ^3^ ^]^ However, on the premise of further improving the efficiency of the Fenton‐like reaction, reducing the amount of TMs is still necessary to reduce the cost and avoid secondary pollution caused by metal ion leaching.^[^ ^4^ ^]^

By dispersing the active metal component into the atomic levels, single‐atom catalysts (SACs) exhibit maximum utilization efficiency of metal atoms, precisely tunable local coordination environment, and strong metal‐support interactions.^[^ ^5^ ^]^ Moreover, the homogeneous structure of SACs provides a promising platform for clarifying the structure‐activity relationship and the fundamental mechanism of interfacial reactions at the molecular and atomic levels.^[^ ^6^ ^]^ The unprecedented improvements in the catalytic properties of SACs have sparked significant research interest in electrocatalysis,^[^ ^7^ ^]^ energy applications,^[^ ^8^ ^]^ thermal catalysis,^[^ ^9^ ^]^ carbon fixation,^[^ ^10^ ^]^ and environmental remediation.^[^ ^11^ ^]^ Based on the construction of SACs, PMS‐based Fenton‐like reaction exhibits excellent water purification performance for the refractory organic pollutants, where SACs inherit the advantages of homogeneous and heterogeneous catalysts, effectively compensating for the drawbacks of TM catalyst in Fenton‐like reaction.^[^ ^4^, ^12^ ^]^

In particular, M‐N‐C SACs feathered with atomically dispersed TM atoms (such as Fe,^[^ ^13^ ^]^ Cu,^[^ ^14^ ^]^ Co,^[^ ^15^ ^]^ and Mn^[^ ^4a^ ^]^) on N‐doped carbon substrates have been extensively studied for PMS‐based Fenton‐like reactions, and the M‐N_x_ sites have been identified as the main active sites for activating PMS. Thus, various attempts have been devoted to increasing the density of M‐N_x_ sites, which significantly improved catalytic activity.^[^ ^16^ ^]^ In a specific range of metal loadings, previous studies have reported a strong positive linear correlation between the reaction rate constant (*k*_obs_) and metal loading.^[^ ^17^ ^]^ In this case, the active metal atoms are far from the neighboring ones. However, when the internal atomically dispersed metals get closer, the significantly enhanced catalytic activity cannot be attributed solely to the increased number of active sites. Because the strong interaction between the adjacent M‐N_x_ sites can alter the electronic structure of the individual sites. For example, Jin et al. demonstrated that site‐to‐site communication (electronic interaction) might appear when the distance of two adjacent Fe atoms is less than about 12 Å.^[^ ^18^ ^]^ Meanwhile, the distance of the neighboring active sites can affect the adsorption and activation modes of the adsorbed molecules. Zhou et al. reported that the O_2_ adsorption modes could transform from a typical Pauling model to a bridge model associated with a change in the active site distance in the oxygen reduction reaction.^[^ ^19^ ^]^ In addition, with the merits of earth abundance, low cost, and low environmental toxicity, Fe─N─C SACs offer an optimal alternative for water purification.^[^ ^20^ ^]^ Therefore, it would be worthwhile to expand the exploration to involve the distance effect of the single‐atom Fe sites on PMS activation.

Herein, we present a comprehensive study on the distance effect in single‐atom Fe catalysts (Fe_SA_‐CN) for PMS‐based Fenton‐like reactions using an integrated experimental and theoretical approach. Typical structures with different distances between adjacent single‐atom Fe sites (*d*_site_) were obtained by varying the density of the single‐atom Fe sites. The catalytic activity of Fe_SA_‐CN exhibited a sharp enhancement at *d*_site_ = 0.5 nm with the assistance of PMS via the electron transfer regime. Along with the decrease in *d*_site_, the electronic communication between the adjacent single‐atom Fe active sites altered the electronic structure of the individual Fe atoms. Moreover, the proximity of the Fe sites enabled the PMS adsorption in a two‐site pathway, which enhanced the electron transfer and promoted PMS decomposition. The PMS decomposition products (OH^−^ and SO_4_^2−^) are readily desorbed from the individual single‐atom Fe sites with the adsorption of pollutants, respectively. The strong adsorption and activation processes of PMS and the easy desorption process of the PMS decomposition products together maintained efficient contaminant removal. Moreover, the Fe_SA_‐CN/PMS system exhibited excellent anti‐interference performance for different anions and aquatic systems and good durability in continuous‐flow experiments, indicating its great potential for water treatment applications. An in‐depth understanding of the distance effect could contribute to the potential application of SACs in water treatment to remove refractory organic pollutants.

## 2 Results and Discussion

**Figure 1:**
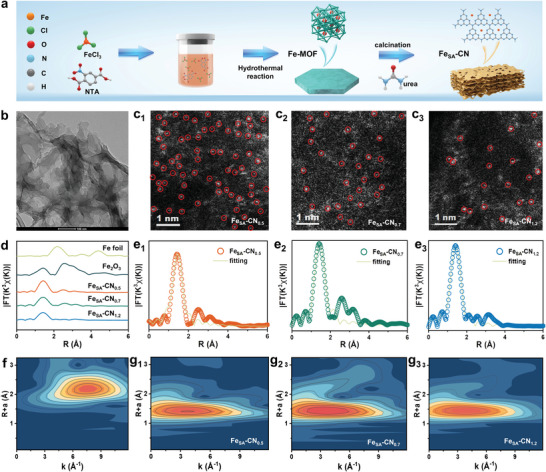
a) Schematic illustration of the synthesis process; b) HRTEM image; c) AC‐HAADF‐STEM images of Fe_SA_‐CN SACs; d) FT‐EXAFS spectra of the Fe foil, Fe_2_O_3_, and Fe_SA_‐CN SACs; e) corresponding EXAFS R‐space fitting curves of Fe_SA_‐CN SACs; WT‐EXAFS spectra of f) Fe foil and g) Fe_SA_‐CN SACs.

As schematically illustrated in **Figure** **1**a, single‐atom Fe catalysts (Fe_SA_‐CN SACs) with three *d*_sites_ values were prepared by the pyrolysis of a mixture of a Fe‐based metal‐organic framework (Fe‐MOF) which is often used in catalyst synthesis^[^ ^21^ ^]^ and urea, followed by acid etching to remove the iron oxide. Due to the N pots with abundant lone‐pair electrons in the graphitic carbon nitride (g‐C_3_N_4_) framework, Fe atoms can be solidly bonded to g‐C_3_N_4_ in a single‐atom distribution. The Fe content of Fe_SA_‐CN SACs was determined to be 0.5, 0.9, and 1.1 wt.% using inductively coupled plasma‐optical emission spectrometry (ICP‐OES), respectively. The X‐ray diffraction (XRD) patterns of Fe_SA_‐CN SACs exhibit two typical peaks of g‐C_3_N_4_ (UCN) at ≈12.7° and 27.5° for the (100) and (002) crystal planes,^[^ ^22^ ^]^ with no peaks assigned to Fe nanoparticles and oxides (Figure S1, Supporting Information). Owing to the strengthening effect of metals on the polymerization of carbon‐based materials,^[^ ^23^ ^]^ Fe_SA_‐CN SACs with low Fe content (0.5 and 0.9 wt.%) show higher crystallinity than pure g‐C_3_N_4_ and Fe_SA_‐CN with a high Fe content (1.1 wt.%). The Fourier‐transform infrared (FTIR) spectra of Fe_SA_‐CN SACs and UCN show similar breathing modes of the tri‐s‐triazine units and typical stretching vibration modes of C═N and C─N heterocycles at ≈809 and 1200–1600 cm^−1^,^[^ ^24^ ^]^ which indicates Fe_SA_‐CN SACs maintain the fundamental configuration of g‐C_3_N_4_ (Figure S2, Supporting Information).

High‐resolution transmission electron microscopy (HRTEM) images and element mapping images based on energy‐dispersive X‐ray (EDX) spectroscopy show that Fe atoms are uniformly distributed on the curled 2D g‐C_3_N_4_ nanosheets (Figure 1b; Figure S3, Supporting Information). The aberration‐corrected high‐angle annular dark‐field scanning transmission electron microscopy (AC‐HAADF‐STEM) images further display uniformly distributed bright spots on the g‐C_3_N_4_ support corresponding to atomically dispersed single Fe atoms, which permits the analysis of the average *d*_site_ values using the computational statistics (Figure 1c; Figure S4, Supporting Information). The distance values of Fe atoms are normal distribution, and the average *d*_site_ value in Fe_SA_‐CN (1.1 wt.%) is 0.5 nm, and the *d*_site_ value increases to 0.7 and 1.2 nm when the Fe loading decreases to 0.9 and 0.5 wt.% (Figure S4, Supporting Information). The coordination environment of Fe_SA_‐CN was further analyzed by X‐ray absorption fine structure (XAFS) spectroscopy. The normalized Fe K‐edge X‐ray absorption near‐edge structure (XANES) spectra of the three Fe_SA_‐CN SACs generally present similar characteristics to those of the reported Fe‐N_4_ configuration structure,^[^ ^25^ ^]^ and the distinct K‐edge absorption of the Fe_SA_‐CN SACs at ca. 7120 eV between those of Fe foil and Fe_2_O_3_ indicates that the valence state of the Fe atoms is between +2–+3 (Figure S5, Supporting Information).^[^ ^26^ ^]^ The Fourier transform extended X‐ray absorption fine structure (FT‐EXAFS) spectra of Fe_SA_‐CN SACs (Figure 1d) present only one prominent peak at approximately 1.5 Å (without phase shift) in the R space corresponding to the Fe─N scattering path. The absence of the first shell Fe–Fe path at 2.2 Å implies that no Fe clusters are present in Fe_SA_‐CN SACs. Subsequently, the first‐shell theoretical fitting of the EXAFS spectra was conducted to quantify the coordination configuration of Fe atoms in the Fe_SA_‐CN SACs. The Fe─N coordination numbers of Fe_SA_‐CN SACs with different Fe loadings are estimated to be 3.8–4.2 with an average bond length of ca. 2.0 Å, demonstrating that the single Fe atom in Fe_SA_‐CN SACs coordinates with 4 N atoms of g‐C_3_N_4_ (Figure 1e; Table S2, Supporting Information). In addition, in the corresponding wavelet transformation (WT) EXAFS results (Figure 1f,g), distinguished from the Fe foil at k value of 7.6 Å^−1^, Fe_SA_‐CN SACs display a central peak at a k value of ca. 4.0 Å^−1^,^[^ ^27^ ^]^ which can be ascribed to the Fe─N coordination at the first shell.

**Figure 2:**
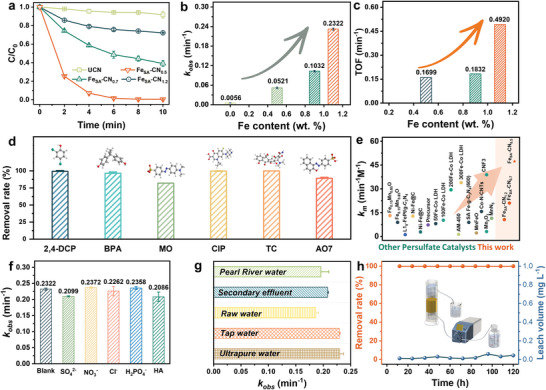
a) 2,4‐DCP degradation curves, b) the corresponding first‐order constants, and c) TOFs in UCN and Fe_SA_‐CN SACs systems; d) degradation of different pollutants in the Fe_SA_‐CN/PMS system; e) comparison of the normalized rate constants of pollutant degradation by PMS activation with the previous reported catalysts, effects of f) anions and HA, and g) various aquatic systems on 2,4‐DCP degradation in the Fe_SA_‐CN/PMS system (Reaction conditions: catalyst dosage = 0.2 g L^−1^, [PMS]_0_ = 0.2 mm, [pollutant]_0_ = 50 µm, [anion]_0_ = 0.2 mm, [HA]_0_ = 2.0 ppm, temperature = 30 °C.); h) performance of Fe_SA_‐CN/PMS system in a continuous‐flow reactor consisting of a Fe_SA_‐CN filled column. (Reaction conditions: catalyst dosage = 100 mg, [PMS]_0_ = 0.2 mm, [2,4‐DCP]_0_ = 50 µm, flow rate = 3.0 mL min^−1^).

2,4‐dichlorophenol (2,4‐DCP), a critical pesticide and pharmaceutical intermediate, was first selected as a probe pollutant to systematically evaluate the catalytic performances of Fe_SA_‐CN SACs (**Figure** **2**a). Compared with Fe‐free g‐C_3_N_4_ (UCN), the Fe_SA_‐CN SACs exhibit significant catalytic activity for 2,4‐DCP degradation with the assistance of PMS. Notably, the introduction of single Fe atoms immediately triggers the PMS activation for rapid 2,4‐DCP degradation, and the apparent inhibition of 2,4‐DCP degradation caused by EDTA as Fe poison further verifies that single‐atom Fe sites are the dominant active sites in the Fenton‐like reaction (Figure S6, Supporting Information). Fe_SA_‐CN SACs with smaller *d*_site_ values exhibit higher observed rate constants (*k*_obs_). In particular, Fe_SA_‐CN_0.5_ can achieve the complete 2,4‐DCP removal within 8 min, and the corresponding *k*_obs_ reach ≈42 times that of UCN (Figure 2b). Remarkably, the doubling of Fe loading (from 0.5 to 0.9 wt.%) leads to the doubling of *k*_obs_ (from 0.0516 to 0.10323 min^−1^). However, a further 0.2‐fold increase of Fe loading (from 0.9 to1.1 wt.%) still results in a doubling of *k*_obs_ (from 0.10323 to 0.2493 min^−1^), and the turnover frequencies (TOFs) of each Fe‐N_4_ site in the Fe_SA_‐CN SACs exhibit a disproportionate sharp enhancement with the increase in Fe loading (Figure 2c). The apparent jump in catalytic activity does not simply arise from the increased number of single‐atom Fe active sites, implying that the distance effect of adjacent Fe atoms may be the key factor in catalytic activity. Fe_SA_‐CN is used instead of Fe_SA_‐CN_0.5_ in the subsequent results and discussion.

The effects of the catalyst dosage and PMS concentration on 2,4‐DCP abatement were further studied (Figures S7 and S8, Supporting Information); when the catalyst dosage exceeds 0.2 g L^−1^, 2,4‐DCP is completely decomposed within 8 min in the Fe_SA_‐CN/PMS (0.2 mm) system. Even when the catalyst dosage is reduced to 0.1 g L^−1^, 96% of 2,4‐DCP can still be removed within 10 min. In the PMS concentration range of 0.2–2.0 mm, the Fe_SA_‐CN/PMS system still maintains almost the same catalytic performance, effectively reducing the PMS resource consumption and subsequent SO_4_^2−^ residue. In addition to 2,4‐DCP, other types of refractory organic pollutants, such as antibiotics (tetracycline/TC and ciprofloxacin/CIP), endocrine disruptors (bisphenol A/BPA), and organic dyes (methyl orange/MO and acid orange 7/AO7), can also be efficiently removed within 15 min in the Fe_SA_‐CN/PMS system (Figure 2d). Furthermore, a normalized rate constant (*k*_n_), obtained by dividing *k*_obs_ by the catalyst dosage and PMS concentration followed by multiplying the contaminant concentration, was employed to evaluate the catalytic performance in various heterogeneous PMS‐based Fenton‐like reaction systems. The *k*_n_ of Fe_SA_‐CN distinctly exceeds those of the other previously reported PMS‐based Fenton‐like catalysts, including the state‐of‐the‐art SACs (Figure 2e; Table S3, Supporting Information).

The anions in water can be transformed into the corresponding anion free radicals with weak oxidation capacity. Nature organic matters (NOMs) can also consume the active species in the AOPs, which inhibits the removal of target pollutants. As shown in Figure 2f, 2,4‐DCP can be efficiently and completely degraded within almost the same *k*_obs_ in the presence of various anions (i.e., SO_4_^2−^, NO_3_^−^, Cl^−^, and H_2_PO_4_^−^) and HA.^[^ ^28^ ^]^ The excellent anti‐interference performance of the Fe_SA_‐CN/PMS system against various anions and NOMs is potent for the treatment of real water systems. Subsequently, the feasibility of the Fe_SA_‐CN/PMS system was evaluated in various aquatic systems (i.e., ultrapure water, tap water, raw water, secondary effluent, and Pearl River water), where Fe_SA_‐CN/PMS system can achieve rapid abatement of the additional 2,4‐DCP within 8–15 min and maintain a high *k*_obs_ (Figure 2g). Moreover, a home‐made continuous‐flow fixed‐bed reactor consisting of a Fe_SA_‐CN_0.5_ filled column was used to evaluate the catalytic performance of the Fe_SA_‐CN/PMS system for continuous water decontamination with a hydraulic retention time of ca. 10 min (Figure 2h). PMS does not directly oxidize 2,4‐DCP (Figure S7, Supporting Information), and the 2,4‐DCP removal rate maintains consistently close to 100% throughout a continuous operating period of 120 h. Meanwhile, the Fe content leached to the effluent is less than 0.05 mg L^−1^, which is far below the national standard of 0.3 mg L^−1^. In addition, the used Fe_SA_‐CN maintains the basic structure, as proved by XRD, FTIR, and HRTEM (Figures S9–S11, Supporting Information). The Fe_SA_‐CN/PMS system demonstrates efficient catalytic performance and stability, exhibiting a great prospect for water treatment applications.

**Figure 3:**
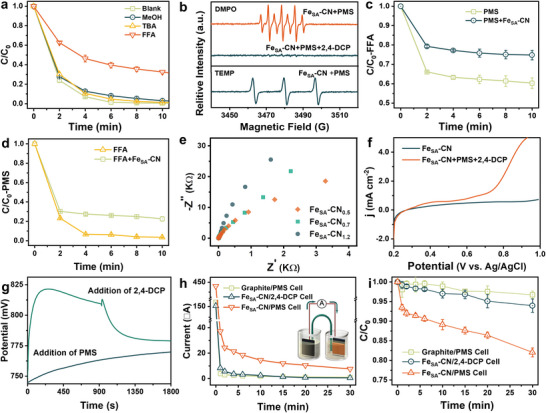
a) Quenching experiments; b) EPR spectra with DMPO/TEMP as the spin‐trapping agents; variations of c) FFA and d) PMS concentrations (Reaction conditions: catalyst dosage = 0.2 g L^−1^, [PMS]_0_ = 0.2 mm, [2,4‐DCP]_0_ = 50 µm, [MeOH]_0_ = [TBA]_0_ = 50 mm, [FFA]_0_ = 5 mm, temperature = 30 °C); e) EIS plots; f) LSV curves; g) open‐circuit potentials; variations of h) current intensity and i) 2,4‐DCP concentration in the GOP system (Reaction conditions: catalyst dosage = 2.0 mg, [PMS]_0_ = 1.0 mm, [2,4‐DCP]_0_ = 400 µm, temperature = 30 °C).

To reveal the reaction mechanisms underlying 2,4‐DCP degradation, reactive oxygen species (ROS) were studied by the quenching experiments. As shown in **Figure** **3**a, the negligible impact on the elimination of 2,4‐DCP by adding methanol (MeOH) and *tert*‐butanol (TBA) excludes the contribution of SO_4_^●−^ and ^●^OH. The significant inhibition on the 2,4‐DCP removal upon adding furfuryl alcohol (FFA) implies that singlet oxygen (^1^O_2_) may be the active species in the Fe_SA_‐CN/PMS system. Furthermore, the electron paramagnetic resonance (EPR) experiments were utilized to further detect the ROS, where DMPO and TEMP were selected as the spin‐trapping reagents for SO_4_^●−^ and ^●^OH, and ^1^O_2_, respectively. As shown in Figure 3b, no typical signals corresponding to DMPO‐SO_4_^●−^ or DMPO‐^●^OH adducts are discerned, which further rules out the presence of SO_4_^●−^ and ^●^OH. The presence of the DMPOX signal indicates the presence of oxidative species within the system that can directly oxidize DMPO.^[^ ^29^ ^]^ The DMPOX signal almost disappears after adding 2,4‐DCP, implying that oxidative species can be consumed for efficient 2,4‐DCP degradation. A triplet EPR signal corresponding to the oxidized TEMP (TEMPO) by ^1^O_2_ is detected in the Fe_SA_‐CN/PMS system, but TEMPO generation could result from the direct oxidation by PMS rather than from the oxidation of ^1^O_2_.^[^ ^30^ ^]^

Subsequently, variations of FFA and PMS concentrations in various systems were detected. As shown in Figure 3c,d, the FFA decay in the PMS alone system and FFA decay in the FFA alone system indicate a direct reaction between FFA and PMS. FFA oxidation and PMS consumption are inhibited with the addition of Fe_SA_‐CN, because Fe_SA_‐CN slows down the depletion of PMS by FFA. Therefore, the inhibition of FFA on the Fe_SA_‐CN/PMS system may be due to the PMS depletion rather than ^1^O_2_ quenching by FFA. To further determine the role of ^1^O_2_, the FFA‐2,4‐DCP competitive dynamics analysis was performed to compare the rate constants of FFA transformation and 2,4‐DCP oxidation in the Fe_SA_‐CN/PMS system. Considering that 2,4‐DCP cannot be destroyed directly by PMS (Figure S7, Supporting Information), the steady‐state concentration of ^1^O_2_ (9.25 × 10^−10^ m) is obtained through the 2,4‐DCP degradation kinetics equation (Text S6, Supporting Information). Based on this, the calculated rate constant of FFA transformation (0.111 s^−1^) is ca. 150 times the actual measured value (7.36 × 10^−4^ s^−1^). Therefore, ^1^O_2_ is excluded as an active species in the Fe_SA_‐CN/PMS system.

In addition to 2,4‐DCP degradation by ROS, the oxidation by high‐valent Fe species and the electron transfer regime were systematically studied in the Fe_SA_‐CN/PMS system. The unique oxygen atom transfer reaction was used to identify the role of high‐valent Fe species, in which methyl phenyl sulfoxide (PMSO) can be selectively oxidized to the corresponding sulfone product [methyl phenyl sulfone (PMSO_2_)], significantly different from SO_4_^●−^/^●^OH‐mediated pathways.^[^ ^31^ ^]^ Compared with the PMS‐alone system, there is no significant increase in PMSO depletion and PMSO_2_ generation in the Fe‐CN SACs/PMS system, indicating the absence of high‐valent Fe species (Figure S12, Supporting Information).

Electrochemical tests were used to detect the electron transfer regime between 2,4‐DCP, Fe_SA_‐CN, and PMS. With the decrease of *d*_site_ value, the Nyquist semicircle of the Fe_SA_‐CN SACs in the electrochemical impedance spectroscopy (EIS) plots becomes smaller, implying less resistance for electron transport, which is more conducive to the occurrence of the electron transfer regime (Figure 3e). In the linear sweep voltammetry (LSV) curves (Figure 3f), the current density of the Fe_SA_‐CN electrode significantly increases with the addition of PMS and 2,4‐DCP, raising the possibility of electron transfer regime in the Fe_SA_‐CN/PMS/2,4‐DCP system. The open‐circuit potential of the Fe_SA_‐CN increases immediately upon the addition of PMS and subsequently decreases upon the addition of 2,4‐DCP, demonstrating that PMS and 2,4‐DCP act as electron acceptors and donors, respectively. To further verify that the electron transfer regime is the dominant process in PMS activation and 2,4‐DCP decomposition, a galvanic oxidation process (GOP) system was developed to separate PMS and 2,4‐DCP into two half‐cells. An agar salt bridge and ampere meter connected the two half‐cells, and Fe_SA_‐CN was coated onto the graphite electrode. The current and the degradation kinetics of 2,4‐DCP were recorded during the reaction. As depicted in Figure 3h,i, no obvious current signal is generated when the graphite electrode is inserted into the PMS cell or when the Fe_SA_‐CN electrode is added to the 2,4‐DCP cell, suggesting that graphite cannot activate PMS, and 2,4‐DCP cannot directly donate electrons to Fe_SA_‐CN. Notably, when the Fe_SA_‐CN electrode is inserted into the PMS cell, 2,4‐DCP without direct contact with Fe_SA_‐CN and PMS undergoes apparent decomposition, and a distinct current signal is recorded, indicating that Fe_SA_‐CN acts as an electron shuttle bridge and further promotes the electron transfer from 2,4‐DCP to PMS. In addition, the main intermediates of 2,4‐DCP in the Fe_SA_‐CN/PMS system were identified by gas chromatography–mass spectrometry (GC–MS). The major intermediate is 2‐chloro‐1,4‐benzoquinone, which is consistent with the results of a study on non‐radical electron transfer process (Figure S13, Supporting Information). The above results collectively demonstrate that the electron transfer regime is the dominant oxidation process for PMS activation and 2,4‐DCP degradation, which explains the excellent anti‐interference performance of the Fe_SA_‐CN/PMS system for various anions and aquatic systems.

**Figure 4:**
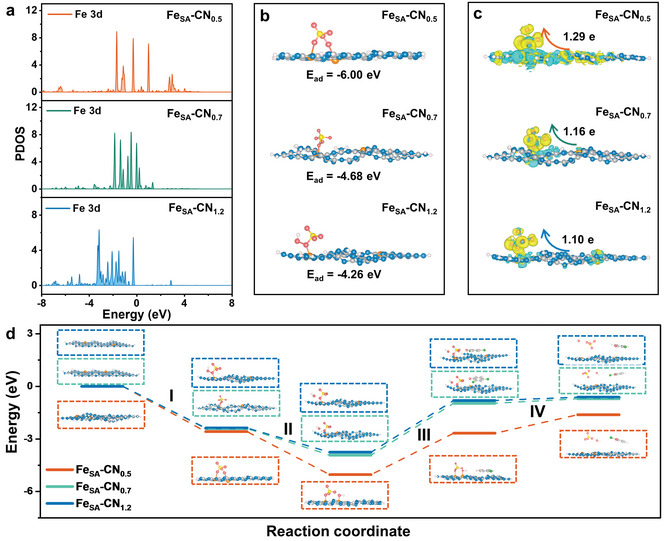
a) PDOS of the Fe_SA_‐CN SACs; b) PMS adsorption models, c) electron density difference for PMS adsorption and the corresponding charge transfer based on Bader charge, and d) reaction pathway of PMS activation and 2,4‐DCP decomposition on the Fe_SA_‐CN SACs.

In order to understand the differences in PMS activation by Fe_SA_‐CN SACs with different *d*_site_ values, density functional theory (DFT) calculations were subsequently performed. Three models of two Fe‐N_4_ sites anchored on g‐C_3_N_4_ with different *d*_site_ are constructed (Figure S14, Supporting Information). Compared with Fe_SA_‐CN_0.7_ and Fe_SA_‐CN_1.2_, the g‐C_3_N_4_ skeleton structure of Fe_SA_‐CN_0.5_ is relatively cracked, consistent with the difference in crystallinity in the XRD results (Figure S1, Supporting Information). The distance predicted by DFT agrees well with the statistical results of the AC‐HAADF‐STEM (Figure S4, Supporting Information). The partial density of states (PDOS) was calculated to reveal the variation of *d* orbitals of Fe in the Fe‐N_4_ models. The band near the Fermi level can overlap with the *p*_z_ orbital of oxygen‐related molecules to form the strong σ bond.^[^ ^32^ ^]^ As shown in **Figure** **4**a, the Fe d‐band center is upshifted and gradually approaches the Fermi level (*E*_f_) with the decrease of *d*_site_ values, which indicates that the proximity of the adjacent Fe atoms results in effective electronic communication. The upshift of the d‐band center reduces the number of electrons entering the final antibonding orbital, thus facilitating PMS activation.

Furthermore, the PMS adsorption models on Fe_SA_‐CN SACs with *d*_site_ = 0.5, 0.7, and 1.2 nm were constructed (Figure 4b; Figure S15, Supporting Information). Fe_SA_‐CN_0.5_ can simultaneously adsorb two O atoms of PMS through two adjacent Fe atoms, while Fe_SA_‐CN_0.7_ and Fe_SA_‐CN_1.2_ can only achieve single‐site adsorption of PMS because of the large distance between the Fe atoms. Consequently, the PMS adsorption energy on Fe_SA_‐CN_0.5_ is significantly greater than those on Fe_SA_‐CN_0.7_ and Fe_SA_‐CN_1.2_, which is consistent with the changing trend of the d‐band center. Large adsorption energy is conducive to PMS activation, but excessively strong adsorption can lead to difficulty in the desorption of PMS intermediates and further poisoning of the active sites,^[^ ^33^ ^]^ which is further discussed in the following section. Based on the optimal PMS adsorption models, the charge density difference was used to analyze the electron transfer process between Fe_SA_‐CN and PMS (Figure 4c). In the three models, the electron accumulation region is mainly concentrated on PMS, implying that Fe_SA_‐CN with different *d*_site_ values can donate electrons to PMS. However, when the *d*_site_ is reduced to 0.5 nm, a wider range of electron depletion region appears on the Fe_SA_‐CN_0.5_ surface, which is more conducive to triggering the electron transfer process. Furthermore, the increased Bader charge values of PMS adsorbed on the Fe_SA_‐CN SACs (*d*_site_ = 0.5, 0.7, and 1.2 nm) are 1.29, 1.16, and 1.10 e respectively, demonstrating that the adjacent Fe atoms in Fe_SA_‐CN_0.5_ can collectively transfer more electrons to PMS for its activation (Figure 4c). Meanwhile, the bond length of PMS peroxy bond in the three models is 1.51, 1.61, and 2.75 Å (Table S4 Supporting information), respectively, and the longer bond lengths are more favorable for PMS activation.^[^ ^4a^ ^]^ Furthermore, the energy diagrams of PMS dissociation on the Fe_SA_‐CN SACs were analyzed. The energy barrier of PMS dissociation on Fe_SA_‐CN SACs is apparently reduced along with the decrease of *d*_site_ value, which indicates that the PMS dissociation to OH^−^ and SO_4_^2−^ on Fe_SA_‐CN_0.5_ is more advantageous in terms of energy variation (Figure S16, Supporting Information).

To better understand the interaction mechanism between Fe_SA_‐CN SACs and the adsorbates (i.e., PMS and 2,4‐DCP), the energy diagram for PMS activation and 2,4‐DCP decomposition was calculated and shown in Figure 4d. First, PMS is adsorbed on the surface of Fe_SA_‐CN SACs, where PMS is adsorbed on the two adjacent Fe sites of Fe_SA_‐CN_0.5_ and on the single Fe site of Fe_SA_‐CN_0.7_ and Fe_SA_‐CN_1.2_ (step I). Then, the adsorbed PMS accepts electrons and dissociates into OH^−^ and SO_4_^2−^. Notably, OH^−^ and SO_4_^2−^ are adsorbed on the two individual Fe sites respectively, which effectively overcomes the difficulty of the intermediate desorption caused by the two‐site pathway. The effective desorption of OH^−^ and SO_4_^2−^ is conducive to the re‐exposure of the Fe active sites and promotes the continuous catalytic reaction. Subsequently, the adsorption of 2,4‐DCP molecule donates electrons to Fe_SA_‐CN SACs (step III), which is the rate‐limiting step of the whole reaction. The energy barrier of Fe_SA_‐CN_0.5_ system in step III is 2.37 eV, which is smaller than those of Fe_SA_‐CN_0.7_ and Fe*_SA_*‐CN_1.2_ systems (2.96 and 3.01 eV), indicating that a more efficient electron transfer regime occurs on Fe_SA_‐CN_0.5_ system. Finally, the OH^−^ and SO_4_^2−^ desorb from the catalyst surface to the solution (step IV). Therefore, by modulating the electronic structure of the active sites and the adsorption/desorption modes of the reactants, the distance effect significantly affects the electron transfer regime among SACs, PMS, and pollutants.

## 3 Conclusion

Increasing the density of active sites in single‐atom Fe catalysts using earth‐abundant elements is an efficient approach to enhance PMS activation for decomposing emerging organic pollutants. However, the excellent catalytic activity is not entirely due to the increased number of single‐atom Fe active sites. This study emphasizes the existence and importance of the distance effect with the proximity of adjacent Fe atoms in a single‐atom Fe catalyst (Fe_SA_‐CN). The proximity of the adjacent Fe atoms can alter the electronic structure of individual Fe atoms to improve the activation of the adsorbed molecules. Moreover, the adjacent single‐atom Fe sites can collectively adsorb PMS molecules with a two‐site pathway, and the PMS decomposition products (OH^−^ and SO_4_^2−^) achieve single‐site desorption from the individual Fe atoms with the adsorption of 2,4‐DCP. Therefore, the distance effect between adjacent single‐atom Fe sites facilitates efficient contaminant remediation through an electron transfer regime. Moreover, the Fe_SA_‐CN/PMS system exhibited excellent anti‐interference performance for different anions and aquatic systems and good durability in continuous‐flow experiments, indicating its great potential for water treatment applications. Thus, this proof‐of‐concept study reveals the variation in catalytic behavior stemming from the distance effect and contributes to the design of highly efficient SACs for practical applications in water treatment.

## Conflict of Interest

The authors declare no conflict of interest.

## Supporting information

Supporting Information

## Data Availability

The data that support the findings of this study are available from the corresponding author upon reasonable request.
