# Supplementary material for: Understanding the Distance Effect of the Single‐Atom Active Sites in Fenton‐Like Reactions for Efficient Water Remediation
Source: Adv Sci (Weinh). 2024 Jan 15;11(12):2307151. doi: 10.1002/advs.202307151 (PMC10966520; doi:10.1002/advs.202307151)
Supplement: Supplementary file 1 — Supporting Information [file ADVS-11-2307151-s001.pdf]

ADVANCED SCIENCE
Open Access

Supporting Information

for *Adv. Sci.*, DOI 10.1002/advs.202307151

Understanding the Distance Effect of the Single-Atom Active Sites in Fenton-Like Reactions for Efficient Water Remediation

*Shuaiqi Zhang, Zhicong Lu, Chun Hu and Fan Li**

Supporting Information

Understanding the Distance Effect of the Single-Atom Active Sites in Fenton-Like Reactions for Efficient Water Remediation

Shuaiqi Zhang, Zhicong Lu, Chun Hu, and Fan Li*

Key Laboratory for Water Quality and Conservation of the Pearl River Delta, Ministry of Education, Institute of Environmental Research at Greater Bay, Guangzhou University, Guangzhou, 510006, China

*Corresponding author

Fan Li, lifan@gzhu.edu.cn, ORCID ID: 0000-0001-5801-127X

This supplementary material includes 33 pages (including this page), 8 texts, 16 figures, and 3 tables.

List of Supporting Information

Text S1. Chemicals and materials.

Text S2. Preparation of catalysts.

Text S3. Characterizations.

Text S4. Evaluation of the catalytic performance.

Text S5. Details of the DMPO- and TEMP-trapping EPR analysis.

Text S6. 2,4-DCP-FFA competition kinetics for Fe_{SA}-CN/PMS system.

Text S7. Details of electrochemical test.

Text S8. Details of theoretical calculations.

Figure S1. XRD patterns of the Fe_{SA}-CN SACs.

Figure S2. FTIR spectra of the Fe_{SA}-CN SACs.

Figure S3. Element mapping images based on EDX spectroscopy of (a) Fe_{SA}-$CN_{0.5}$, (b) Fe_{SA}-$CN_{0.7}$, and (c) Fe_{SA}-$CN_{1.2}$.

Figure S4. AC-HAADF-STEM images and statistic distributions of average d_{site} between the adjacent Fe atoms in (a) Fe_{SA}-$CN_{0.5}$, (b) Fe_{SA}-$CN_{0.7}$, and (c) Fe_{SA}-$CN_{1.2}$.

Figure S5. Normalized Fe K-edge XANES spectra of Fe foil, Fe_2O_3, and Fe_{SA}-CN SACs.

Figure S6. Effect of EDTA on 2,4-DCP degradation in the Fe_{SA}-CN/PMS system.

Figure S7. Effect of catalyst dosage on 2,4-DCP degradation in the Fe_{SA}-CN/PMS system.

Figure S8. Effect of PMS concentration on 2,4-DCP degradation in the Fe_{SA}-CN/PMS system.

Figure S9. XRD pattern of the used Fe_{SA}-CN.

Figure S10. FTIR spectrum of the used Fe_{SA}-CN.

Figure S11. Element mapping images based on EDX spectroscopy of the used Fe_{SA}-CN.

Figure S12. Selective transformation of PSMO into $PMSO_2$.

Figure S13. GC/MS chromatograms of 2,4-DCP after oxidation with Fe_{SA}-$CN_{0.5}$.

Figure S14. Models of Fe_{SA}-$CN_{0.5}$, Fe_{SA}-$CN_{0.7}$, and Fe_{SA}-$CN_{1.2}$.

Figure S15. PMS adsorption models on Fe_{SA}-$CN_{0.5}$, Fe_{SA}-$CN_{0.7}$, and Fe_{SA}-$CN_{1.2}$.

Figure S16. Calculated energy diagrams of PMS dissociation on Fe_{SA}-$CN_{0.5}$, Fe_{SA}-$CN_{0.7}$, and Fe_{SA}-$CN_{1.2}$.

Table S1. Molecular structures of organic compounds and HPLC settings.

Table S2. EXAFS fitting parameters at the Fe K-edge for different samples.

Table S3. Comparison of the normalized rate constants of pollutant degradation by PMS activation with the previous reported catalysts.

Table S4. PMS adsorption modeling peroxide bond lengths in different samples.

Text S1. Chemicals and materials.

Sodium thiosulfate ($Na_2S_2O_3$, 99.0%), sodium bicarbonate ($NaHCO_3$, 99.8%), furfuryl alcohol ($C_5H_6O_2$, FFA, 98.0%), and tert-Butanol ($C_4H_{10}O$, TBA, 99.0%) were obtained from Shanghai Macklin Biochemical Co., Ltd. Methanol (CH_3OH, MeOH, 99.9%), 2,4-dichlorophenol ($C_6H_4Cl_2O$, 2,4-DCP, 98.0%), bisphenol A ($C_{15}H_{16}O_2$, BPA, 98.0%), 2-propanol ($(CH_3)_2CHOH$, IPA, 99.8%), ciprofloxacin ($C_{17}H_{18}FN_3O_3$, CIP, 99.0%), tetracycline ($C_{22}H_{24}N_2O_8$, TC, 96.0%), methyl orange ($C_{14}H_{14}N_3NaO_3S$, IND), acid Orange 7 ($C_{16}H_{11}N_2NaO_4S$, 99%), nafion perfluorinated resin ($C_9HF_{17}O_5S$, RG), potassium monopersulfate triple salt ($(KHSO_5$•$0.5KHSO_4$•$0.5K_2SO_4)$, PMS, 42.8 – 46.0%), 2,2,6,6-tetramethyl-4-piperidinol ($C_9H_{19}NO$, TEMP, 98.0%), and sodium Sulfate (Na_2SO_4, 99.0%) were obtained from Adamas Reagent Co., Ltd. 5,5-dimethyl-1-pyrroline *N*-oxide ($C_6H_{11}NO$, DMPO) was purchased from DOJINDO, Japan. Nitroterephthalic acid ($C_8H_5NO_6$, 98%), and humic acid (HA, 90.0%) were obtained from Shanghai Aladdin Biochemical Technology Co., Ltd. N, N-Dimethylformamide ($HCON(CH_3)_2$, 99.5%), urea (NH_2CONH_2, 99%), potassium iodide (KI, 99%), ethylene diamine tetraacetic acid ($C_{10}H_{16}N_2O_8$, EDTA, 99%) and ferric chloride ($Fe(Cl)_3$, 99.0%) were obtained from General-reagent® Co., Ltd. Dichloromethane (CH_2Cl_2, 99.5%) was purchased from Sinopharm Chemical Reagent Co. Ltd, China. All solutions were prepared using ultrapure water (Milli-Q, 18.2 MΩ cm) produced from a purification system.

Text S2. Preparation of catalysts.

The copolymerization of Fe-MOF and urea carried out the preparation of single-atom Fe catalysts (Fe_{SA}-CN SACs). Briefly, 2.5 mmol nitroterephthalic acid ($C_8H_5NO_6$, NTA) and 5.0 mmol ferric chloride ($FeCl_3$) were dissolved in 30 mL N, N-dimethylformamide (DMF) consecutively. After stirring at 30°C for 60 min, the resulting mixture solution was transferred to a 50 mL Teflon-lined steel autoclave and placed in an oven at 110°C for 24 h. After the oven temperature dropped to room temperature, the residual product (Fe-MOF) obtained by centrifugation was washed three times with DI water and ethanol and dried overnight at 60°C in vacuum. Subsequently, a certain amount of Fe-MOF and urea were thoroughly mixed and transferred to a quartz boat in a tube furnace. The sample was pyrolyzed at 550°C for 4 h at an ascending 5°C/min temperature under an N_2 atmosphere. The yellow solid obtained at room temperature was thoroughly ground and stirred in a 0.1 M H_2SO_4 solution for 6 h to remove the aggregated metal clusters and particles. The residue was washed with DI water until the filtrate became neutral and then dried overnight at 60°C in vacuum. In addition, Fe-free g-C_3N_4 (UCN) was prepared by direct calcination of urea. Fe_{SA}-CN SACs with different d_{site} values were synthesized by varying the amount of Fe-MOF.

Text S3. Characterizations.

The Fe concentration in the catalyst was measured using inductively coupled plasma-optical emission spectrometry (ICP-OES) (Avio 200, PerkinElmer). X-ray diffraction (XRD) analysis was performed on a PANalytical X-ray diffractometer (PW3040/60) using Cu Kα radiation at a scanning angle (2θ) of 10°-80°. FTIR spectra analysis was recorded on a Nicolet iS10 Fourier Transform Infrared Spectroscopy spectrometer (Thermo Fisher Scientific Inc., USA) in the range of 4000 – 400 cm^{-1}. High-resolution transmission electron microscopy (HRTEM) images and energy-dispersive X-ray spectroscopy (EDX) were obtained using FEI Talos F200S. Aberration-corrected high-angle annular dark-field scanning transmission electron microscopy (HAADF-STEM) images were recorded using a Titan Themis Z. JEOL ARM200CF. Electron paramagnetic resonance (EPR) spectra were collected using a Bruker A300-10/12 electron paramagnetic resonance spectrometer. The X-ray absorption spectra (XAS) including X-ray absorption near-edge structure (XANES) and extended X-ray absorption fine structure (EXAFS) of the samples at Fe K-edge (7709 eV were collected at the Singapore Synchrotron Light Source (SSLS) center, where a pair of channel-cut Si (111) crystals was used in the monochromator. The Fe K-edge XANES data were recorded in a transmission mode. Fe foil was used as references. Athena and Artemis software were used to process and analyze the data. The PMS concentration was measured using a spectrophotometric method on a Hach DR 6000 UV−vis spectrometer. The electrochemical measurements were performed using a standard three-electrode cell system on a Zahner electrochemical workstation. ITO conductive glass coated with catalyst, Pt wire, and Ag/AgCl electrode were employed as the working, counter, and reference electrodes. Gas chromatography-mass spectrometry (GC-MS) was used to analyze the intermediate products. For volatile product identification, 50 mL of the sample was extracted with 2 mL of dichloromethane, and the extract was analyzed on a Shimadzu GC/MS-QP2020 NX in EI mode using a full scan range of 40-400 m/z (DB-1701 column, 30 m × 0.25 mm × 0.25 μm; injector 270°C, oven 50°C held for 2 min, and then ramped to 250 °C at 5 °C min^{-1}, and auxiliary 280°C).

Text S4. Evaluation of the catalytic performance.

The degradation of 2,4-DCP which acts as a model organic pollutant was used to evaluate the performance of the prepared catalysts for PMS activation. The experiments were carried out in 50 mL of aqueous solution in a 30 °C water bath with magnetic stirring. Add contaminant (50 μM) and catalyst (0.2 g/L) together to 50 mL of aqueous solution, followed by PMS (0.2 mM) to get the reaction started. At certain intervals, 1 mL of the reaction solution was filtered through a de-tetrafluoroethylene membrane (0.45 μm) and subsequently collected into a liquid chromatography vial for subsequent component analysis. An excess of sodium thiosulfate (Na_2SO_3) solution was added to each liquid chromatography vial to terminate the reaction. The concentration of 2,4-DCP, BPA, CIP, and TC were measured by high-performance liquid chromatography (HPLC, Shimadzu LC-20 AT) equipped with a UV-vis detector. MO and AO7 were measured on a ultraviolet spectrophotometer. The degradation of the above pollutants is consistent with pseudo-first-order kinetic constants and reaction rate constants (k_{obs}) determined by eq. S1

$$\ln \left(\frac{c_t}{c_0}\right) = -k_{obs} \times t \tag{S1}$$

where c_t and c_0 are the pollutant concentration at reaction time (t) and initial moment, respectively.

The turnover frequency (TOF) of the Fe sites of the catalyst can represent the metal site activity of the catalyst, which can be determined by eq. S2.

$$\mathrm{TOF}\ [\mathrm{min}^{-1}] = \frac{\text{Moles of pollutant transformed}}{\text{Moles of active site} \times \text{response time}} = \frac{\Delta n(2,4\text{-DCP})}{n_{metal} \times t} = \frac{\Delta c(2,4\text{-DCP}) \times V \times M_{metal}}{m_0 \times \omega_{metal} \times t} \tag{S2}$$

where Δn(2,4-DCP) is the moles of 2,4-DCP degraded, n_{metal} is the moles of metals sites, t is the reaction time, Δc(2,4-DCP) is the concentration of 2,4-DCP degraded, V is the volume of reaction solution, M_{metal} is the molecular mass of metals, m_0 is the mass of catalyst, and ω_{metal} is the mass fraction of metal in catalyst.

The modified rate constant (k_n) is calculated as eq. S3

$$k_n = \frac{k * [\mathrm{P}]}{[\mathrm{cat.}] * [\mathrm{PS}]} \tag{S3}$$

Where k = pseudo-first-order kinetic constant, [P] = pollutant degradation, [cat.] = catalyst dosage, and [PS] = peroxide concentration.

Text S5. Details of the DMPO- and TEMP-trapping EPR analysis.

After reacting for 5 min, 1.0 mL of the catalyst was withdrawn, and 20 μL of DMPO/TEMP (5.0 M) was added. The suspension was then transferred to a micropipette and packed into an EPR quartz tube for the detection of $SO_4^{\bullet -}$ and $^{\bullet}OH$, and $^1O_2$, Reaction conditions: catalyst dosage = 0.2 g L^{-1}, $[PMS]_0$ = 0.20 mM, $[2,4\text{-}DCP]_0$ = 50 μM (if any), temperature = 30 °C.

Text S6. 2,4-DCP-FFA competition kinetics for Fe_{SA}-CN/PMS system.

2,4-DCP (50 μM) and furfuryl alcohol FFA (5 mM) were added to the Fe_{SA}-CN/PMS system to elucidate the role of singlet oxygen for 2,4-DCP degradation. The first-order kinetic constants for 2,4-DCP (k_{1D} = 4.72×10^{-3} s^{-1}) and FFA (k_{1F} = 7.36×10^{-4} s^{-1}) are calculated from the reaction kinetics of 2,4-DCP and FFA with $^1O_2$, respectively (Figure S12). Assuming that $^1O_2$ is the main active species for 2,4-DCP degradation, the steady-state concentration of $^1O_2$ ($[^1O_2]_{ss}$) in the Fe_{SA}-CN/PMS system would be 2.01×10^{-10} M obtained from the reaction kinetics of $^1O_2$ with 2,4-DCP (eq. S3). Thus, based on the reaction kinetics of $^1O_2$ with FFA and $[^1O_2]_{ss}$, the theoretical value of the first-order kinetic constant of $^1O_2$ with FFA ($k_{1F(\mathrm{theoretical})}$) was 0.111 M^{-1} s^{-1} (eq. S4), which is approximately 150.8 times higher than the experimental value (k_{1F}). The detailed calculation process is as follows.

$$\frac{d[2,4-DCP]}{dt} = -k_{(1O2,\ 2,4-DCP)} \times [^1O_2]_{ss} \times [2,4\text{-DCP}] = -k_{1D} \times [2,4\text{-DCP}] \quad \text{(S3)}$$

$$k_{1D} = 4.72 \times 10^{-3}\ \mathrm{s^{-1}}$$

$$k_{(1O2,\ 2,4\text{-DCP})} = 5.10 \times 10^6\ M^{-1}s^{-1}$$

$$[^1O_2]_{ss} = \frac{k1D}{\mathrm{k(1O2,2,4-DCP)}} = 9.25\times10^{-10}\ \mathrm{M}$$

$$\frac{d[FFA]}{dt} = -k_{(1O2,FFA)} \times [^1O_2]_{ss} \times [\mathrm{FFA}] = -k_{1F} \times [\mathrm{FFA}] \quad \text{(S4)}$$

$$k_{1F} = 7.36 \times 10^{-4}\ \mathrm{s^{-1}}$$

$$k_{(1O2,\mathrm{FFA})} = 1.20 \times 10^8\ \mathrm{M}^{-1}s^{-1}$$

$$k_{1F(\mathrm{theoretical})} = k_{(1O2,\ \mathrm{FFA})} \times [^1O_2]_{ss} = 0.111\ \mathrm{s^{-1}}$$

$$\frac{k1F(\mathrm{theoretical})}{k1F} = 150.8$$

Text S7. Details of electrochemical test.

The electrochemical properties of Fe_{SA}-CN SACs were tested using a Zahner electrochemical workstation (zahner zennium pro) in standard three-electrode mode, where Ag/AgCl was used as the reference electrode, platinum sheet as the counter electrode, and 0.1 M Na_2SO_4 solution (50 mL) as the electrolyte solution. 500 μL of catalyst solution (10 g L^{-1}) and nafion solution (20 wt%) mixed solution was deposited on the indium-tin-oxide (ITO) conductive glass electrode in 1×1 cm^2 size. The line sweep voltammetry plots were recorded at a scan rate of 10 mV s^{-1} within a range of 200 mV to 1000 mV. Electrochemical impedance spectroscopy (EIS) measurements were performed at the open circuit potential using sinusoidal signal over frequencies ranging from 0.01 Hz to 10^6 Hz.

Text S8. Details of theoretical calculations.

All the calculations are performed in the framework of the density functional theory with the projector augmented plane-wave method, as implemented in the Vienna ab initio simulation package.[1] The generalized gradient approximation proposed by Perdew-Burke-Ernzerhof (PBE) is selected for the exchange-correlation potential.[2] The cut-off energy for plane wave is set to 450 eV. The energy criterion is set to 10^{-4} eV in the iterative solution of the Kohn-Sham equation. All the structures are relaxed until the residual forces on the atoms have declined to less than 0.02 eV/Å. To avoid interlaminar interactions, a vacuum spacing of 20 Å is applied perpendicular to the slab. The adsorption energy E_{ads} is expressed as

$$\Delta E_{ads} = E_{A+B} - E_A - E_B \qquad \text{(eq. S5)}$$

Where E_{A+B} is the total energy of slab *A* model with molecule *B* adsorption, E_A is the energy of a A slab, and E_B is that for a *B* molecule.

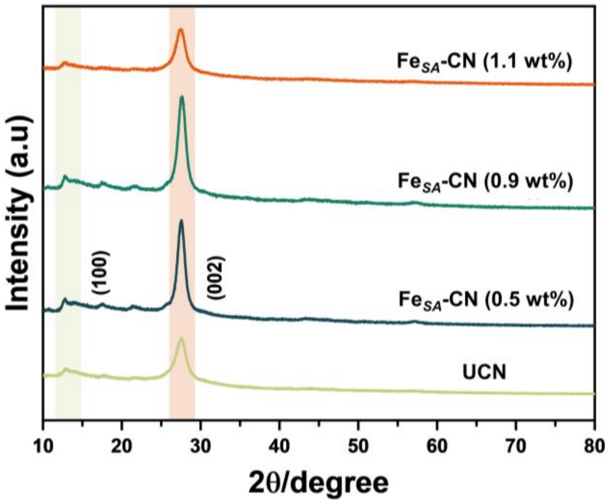

Figure S1. XRD patterns of Fe$_{SA}$-CN SACs.

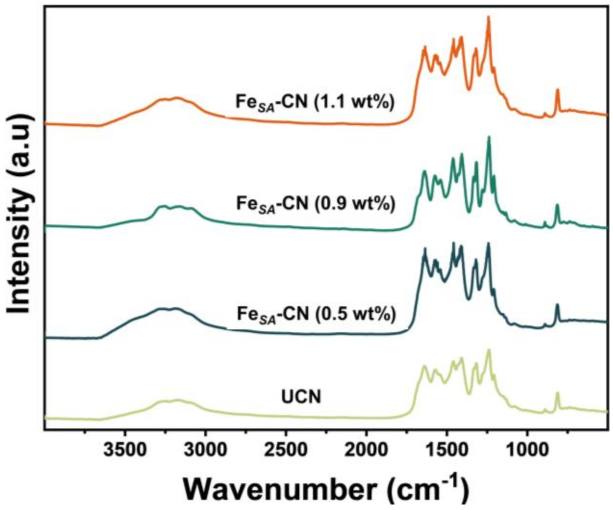

Figure S2. FITR spectra of Fe$_{SA}$-CN SACs.

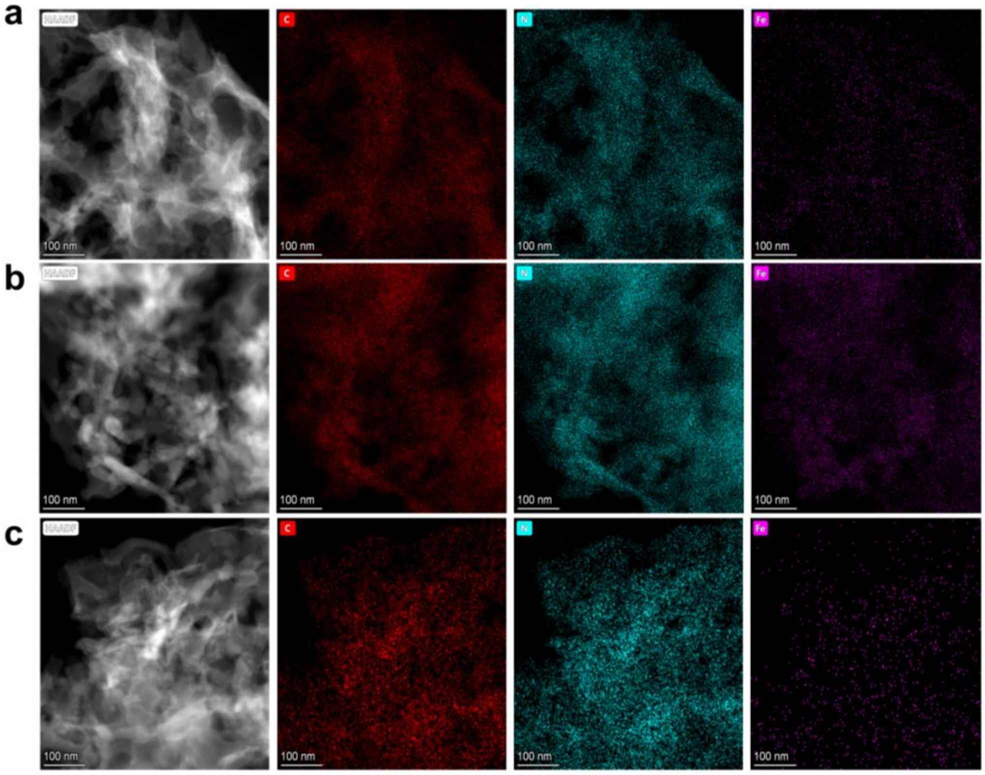

Figure S3. Element mapping images based on EDX spectroscopy of (a) Fe_{SA}-$CN_{0.5}$, (b) Fe_{SA}-$CN_{0.7}$, and (c) Fe_{SA}-$CN_{1.2}$.

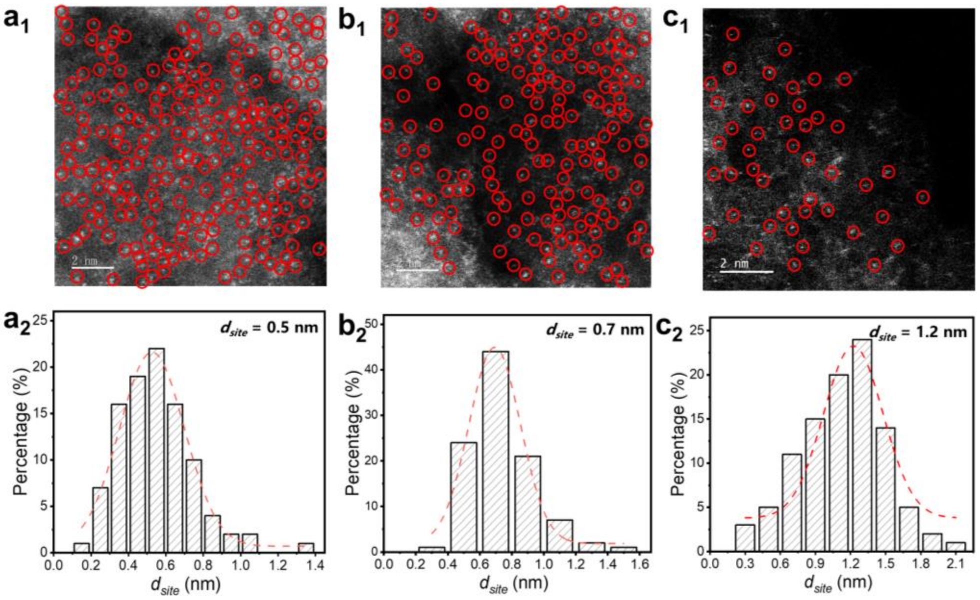

Figure S4. HAADF-STEM images and statistic distributions of average d_{site} between the adjacent Fe atoms in (a) Fe_{SA}-$CN_{0.5}$, (b) Fe_{SA}-$CN_{0.7}$, and (c) Fe_{SA}-$CN_{1.2}$.

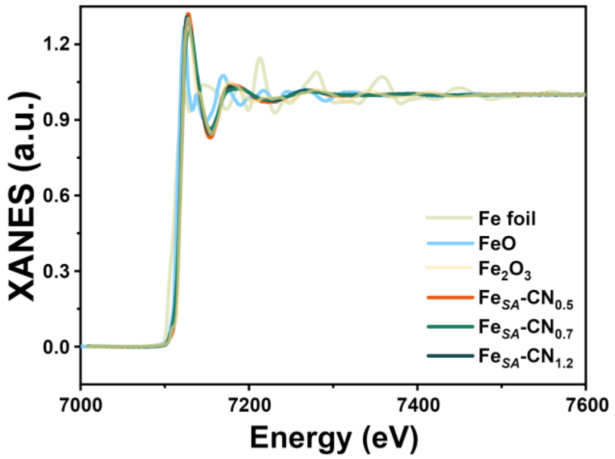

Figure S5. Normalized Fe K-edge XANES spectra of Fe foil, Fe_2O_3, and Fe_{SA}-CN SACs.

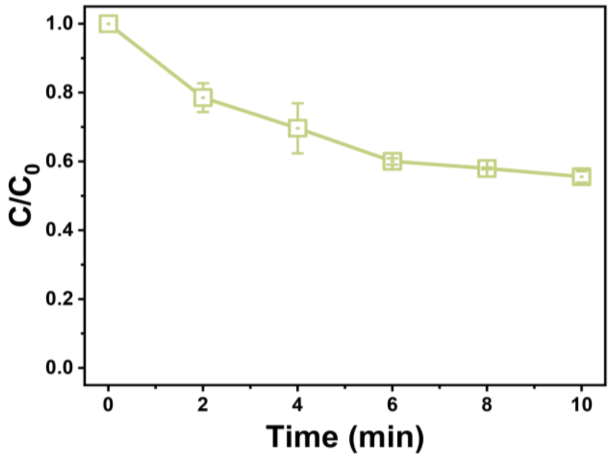

Figure S6. Effect of EDTA on 2,4-DCP degradation in the Fe_{SA}-CN/PMS system. Reaction conditions: catalyst dosage = 0.2 g L^{-1}, $[PMS]_0$ = 0.2 mM, $[2,4\text{-DCP}]_0$ = 50 μM, $[EDTA]_0$ = 1.0 mM, temperature = 30 °C.

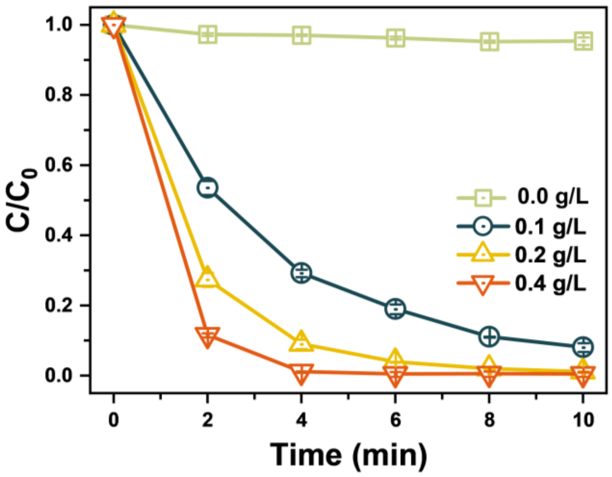

Figure S7. Effect of catalyst dosage on 2,4-DCP degradation in the Fe_{SA}-CN/PMS system. Reaction conditions: $[PMS]_0 = 0.2$ mM, $[2,4\text{-DCP}]_0 = 50$ μM, temperature = 30 ℃.

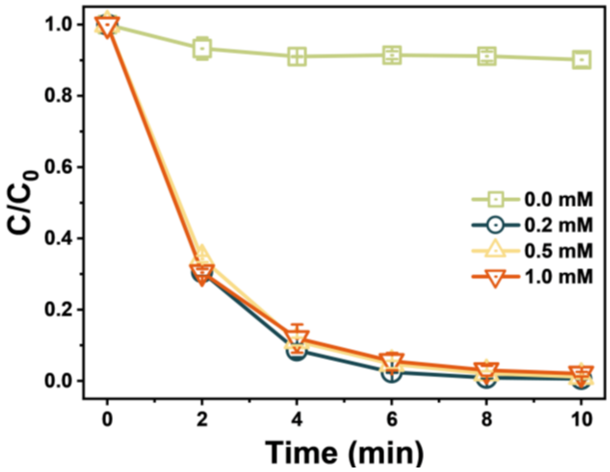

Figure S8. Effect of PMS concentration on 2,4-DCP degradation in the Fe_{SA}-CN/PMS system. Reaction conditions: catalyst dosage = 0.2 g L^{-1}, $[2,4\text{-DCP}]_0$ = 50 μM, temperature = 30 °C.

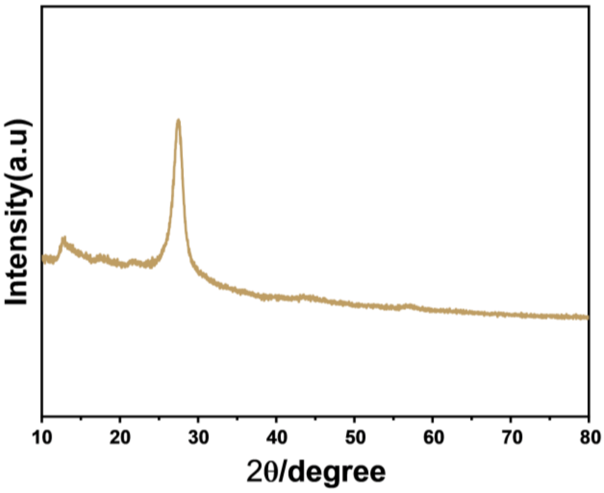

Figure S9. XRD patterns of the used Fe_{SA}-CN.

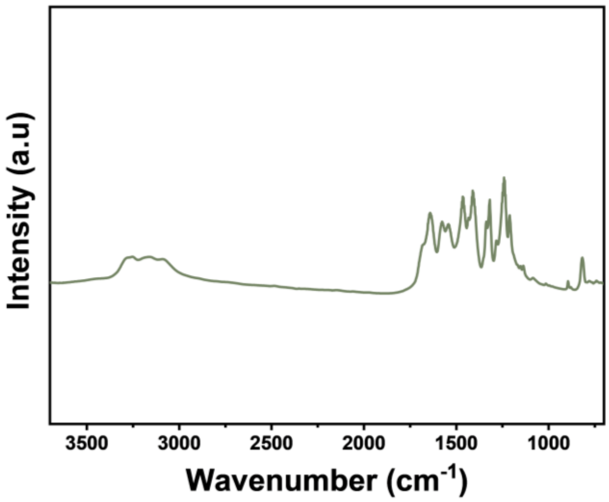

Figure S10. FTIR spectrum of the used Fe_{SA}-CN.

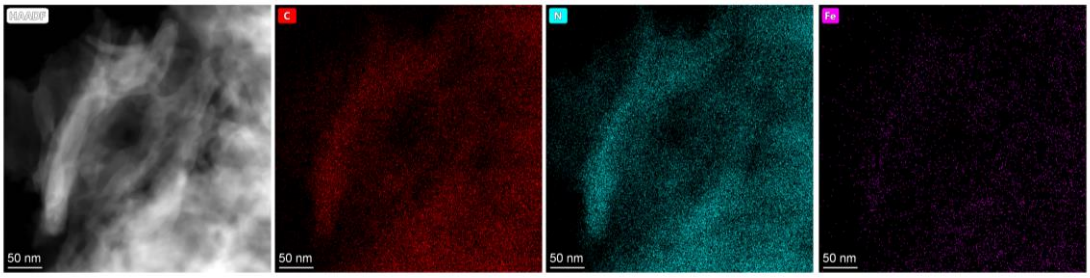

Figure S11. Element mapping images based on EDX spectroscopy of the used Fe_{SA}-CN.

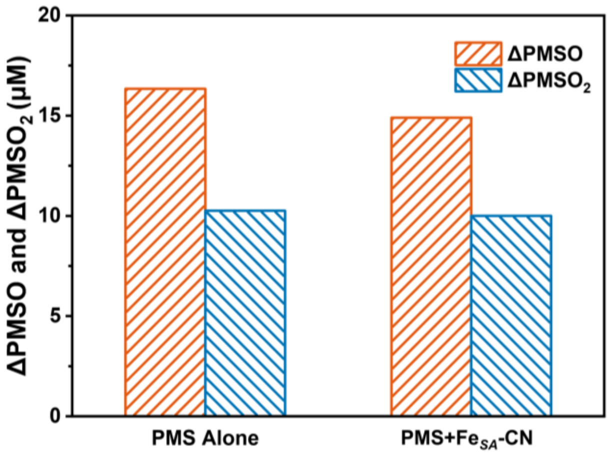

Figure S12. Selective transformation of PSMO into $PMSO_2$. Reaction conditions: catalyst dosage = 0.2 g L^{-1}, $[PMS]_0$ = 0.2 mM, $[PMSO]_0$ = 0.2 mM, temperature = 30 ℃.

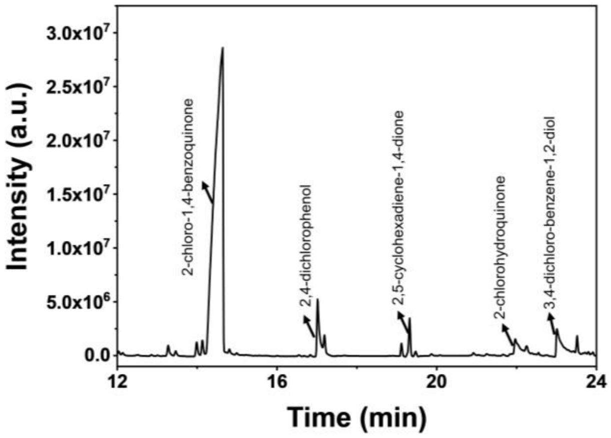

Figure S13. GC/MS chromatograms of 2,4-DCP after oxidation with Fe_{SA}-$CN_{0.5}$.

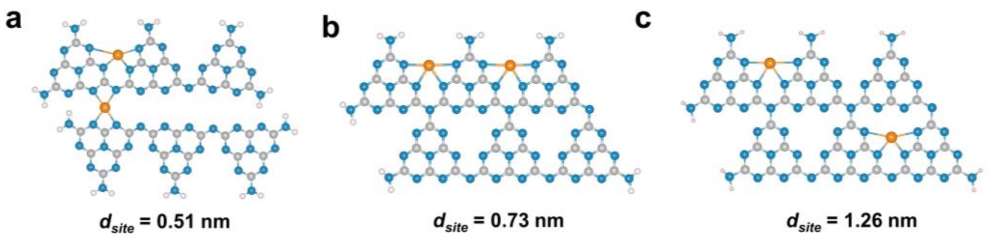

Figure S14. Models of Fe_{SA}-$CN_{0.5}$, Fe_{SA}-$CN_{0.7}$, and Fe_{SA}-$CN_{1.2}$.

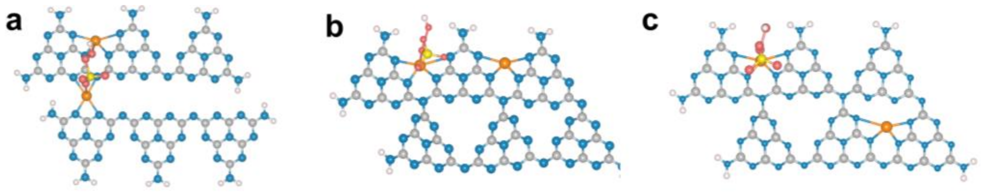

Figure S15. PMS adsorption models on Fe_{SA}-$CN_{0.5}$, Fe_{SA}-$CN_{0.7}$, and Fe_{SA}-$CN_{1.2}$.

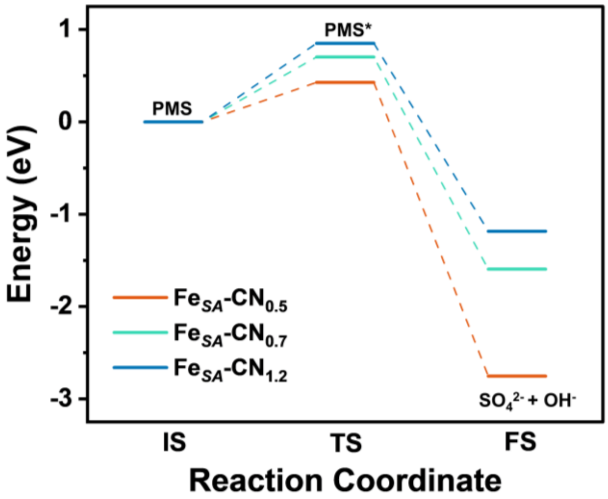

Figure S16. Calculated energy diagrams of PMS dissociation on Fe$_{SA}$-CN$_{0.5}$, Fe$_{SA}$-CN$_{0.7}$, and Fe$_{SA}$-CN$_{1.2}$.

Table S1. Molecular structures of organic compounds and HPLC settings.

Compound	Structure	Detection Wavelength (nm)	Mobile Phase (V/V)
2,4-dichlorophenol (2,4-DCP)	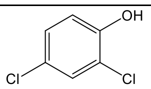	282	methanol : water = 80:20
ciprofloxacin (CIP)		275	acetonitrile : water(0.8% phosphoric acid) = 60:40
bisphenol A (BPA)	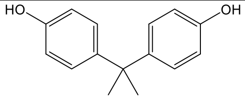	225	methanol : water = 70:30
tetracycline (TC)	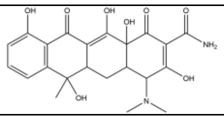	355	Methanol : water(0.8% phosphoric acid) : methanol = 20:60:20
furfuryl alcohol （FFA）	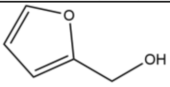	218	acetonitrile : water(0.8% phosphoric acid) = 80:20
methyl phenyl sulfoxide (PMSO)	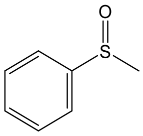	218	acetonitrile : water(0.8% phosphoric acid) = 40:60
methyl phenyl sulfone ($PMSO_2$)	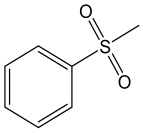	218	acetonitrile : water(0.8% phosphoric acid) = 40:60

Table S2. EXAFS fitting parameters at the Fe K-edge for different samples （S_0^2=0.89）.

Sample	Shell	CN	R(Å)	σ^2 ($\times10^{-3}$ Å^2)	ΔE_0 (eV)	R factor
Fe foil	Fe-Fe	8.0	2.47	6.3	-3.45	0.0096
Fe_{SA}-$CN_{0.5}$	Fe-N	4.2	2.00	5.5	-9.83	0.0175
Fe_{SA}-$CN_{0.7}$	Fe-N	3.9	2.02	6.3	-8.28	0.0139
Fe_{SA}-$CN_{1.2}$	Fe-N	3.8	2.01	7.8	-8.33	0.0150

CN: coordination numbers; R: bond distance; σ^2: Debye-Waller factors; ΔE_0: the inner potential correction. R factor: goodness of fit. S_0^2 was set to 0.89, according to the experimental EXAFS fit of metal foil reference by fixing Fe as the known crystallographic value.

Table S3. Comparison of the normalized rate constants of pollutant degradation by PMS activation with the previous reported catalysts. The normalized rate constant (k_n) model was obtained after multiplying the pseudo-first-order kinetic constants (k_{obs}) by the pollutant concentration and dividing by the catalyst and PMS dosage.

**Catalyst (g L^{-1})**	**PMS (mM)**	**Pollutant (mg L^{-1})**	***k_{obs}* (min^{-1})**	***k_n* (min^{-1} M^{-1})**	**Mechanism**	**Ref.**
Fe_{SA}-$CN_{0.5}$ (0.20)	**0.2**	2,4-DCP (8.15)	0.232	47.3	electron-transfer	This work
Fe_{SA}-$CN_{0.7}$ (0.20)	0.2	2,4-DCP (8.15)	0.103	21.0	electron-transfer	This work
Fe_{SA}-$CN_{1.2}$ (0.20)	0.2	2,4-DCP (8.15)	0.052	10.6	electron-transfer	This work
$Fe_{0.15}Mn_{0.85}O$ (0.04)	0.5	BPA (1.14)	0.230	13.1	electron-transfer	1[3]
$Fe_{0.15}Mn_{0.85}O$ (0.04)	0.5	2,4,6-TCP （0.987）	0.180	8.88	electron-transfer	1[3]
$L1_0$-FePt/g-C_3N_4 (1.0)	2.5	BPA (20)	0.140	1.12	$SO_4^{\bullet-}$	2[4]
Ni-Fe@C (0.10)	0.2	BPA (12.56)	0.225	12.8	electron-transfer	3[5]
Ni-Fe@C (0.10)	0.2	Phenol (9.4)	0.06	2.82	electron-transfer	3[5]
Precursor (0.02)	0.2	BPA (2.0)	0.0145	7.25	$^1O_2$	4[6]
50Fe-Co LDH (0.02)	0.2	BPA (2.0)	0.0161	8.04	$^1O_2$	4[6]
100Fe-Co LDH (0.02)	0.2	BPA (2.0)	0.0205	10.2	$^1O_2$	4[6]
200Fe-Co LDH (0.02)	0.2	BPA (2.0)	0.0587	29.4	$^1O_2$	4[6]
300Fe-Co LDH (0.02)	0.2	BPA (2.0)	0.0678	33.9	$^1O_2$	4[6]
AM-450 (0.4)	1.0	CIP (10)	0.0514	1.29	electron-transfer	5[7]
SA Fe-g-C_3N_4(600) (0.1)	0.5	TC (10)	0.0443	8.86	$^1O_2$ Fe^{IV}=O	6[8]

MnFeO (0.4)	0.325	*p*-ASA (9.9)	0.0285	2.17	direct oxidation	7[9]
CNF3 (0.1)	0.4	4-CP (12.56)	0.155	38.9	Fe^{V}=O	8[10]
Co-N-CNTs (0.1)	1.0	SMX (10)	0.157	15.7	electron-transfer	9[11]
Mn_2O_3 (0.2)	0.2	Phenol (4.7)	0.13	3.055	Mn(IV)	10[12]
MnN_5 (0.5)	1.0	4-CP (10)	0.572	11.44	N_5Mn(IV)=O	11[13]

Table S4. PMS adsorption modeling peroxide bond lengths in different samples.

Catalyst	Bond	Length (Å)
Fe_{SA}-$CN_{1.2}$	O-O	1.51
Fe_{SA}-$CN_{0.7}$	O-O	1.61
Fe_{SA}-$CN_{0.5}$	O-O	2.75

References

[1] G. Kresse, D. Joubert, *Phys. Rev. B* 1999, *59*, 1758.

[2] J. P. Perdew, K. Burke, M. Ernzerhof, *Phys. Rev. Lett.* 1996, *77*, 3865.

[3] K. Z. Huang, H. Zhang, *Env. Sci Technol* 2019, *53*, 12610.

[4] M. Ren, J. Hou, J. Ma, Y. Zhang, M. Liu, X. Tan, P. Zhao, A. Lin, J. Cui, *Sep. Purif. Technol.* 2022, *302*, 122105.

[5] E.-T. Yun, S.-W. Park, H. J. Shin, H. Lee, D.-W. Kim, J. Lee, *Appl. Catal. B Environ.* 2020, *279*, 119360.

[6] L. Wu, Z. Sun, Y. Zhen, S. Zhu, C. Yang, J. Lu, Y. Tian, D. Zhong, J. Ma, *Environ. Sci. Technol.* 2021, *55*, 15400.

[7] Y. Zhao, H. Wang, J. Ji, X. Li, X. Yuan, L. Jiang, J. Yang, Y. Shao, X. Guan, *J. Clean. Prod.* 2022, *362*, 132442.

[8] X. Peng, J. Wu, Z. Zhao, X. Wang, H. Dai, L. Xu, G. Xu, Y. Jian, F. Hu, *Chem. Eng. J.* 2022, *427*, 130803.

[9] M. K. Ke, G. X. Huang, S. C. Mei, Z. H. Wang, Y. J. Zhang, T. W. Hua, L. R. Zheng, H. Q. Yu, *Environ. Sci. Technol.* 2021, *55*, 7063.

[10] H. Li, C. Shan, B. Pan, *Environ. Sci. Technol.* 2018, *52*, 2197.

[11] J. Miao, Y. Zhu, J. Lang, J. Zhang, S. Cheng, B. Zhou, L. Zhang, P. J. J. Alvarez, M. Long, *ACS Catal.* 2021, *11*, 9569.

[12] H. Li, N. Yuan, J. Qian, B. Pan, *Environ. Sci. Technol.* 2022, *56*, 4498.

[13] J. Miao, J. Song, J. Lang, Y. Zhu, J. Dai, Y. Wei, M. Long, Z. Shao, B. Zhou, P. J. J. Alvarez, L. Zhang, *Environ. Sci. Technol.* 2023, *57*, 4266.
